# Microenvironment-Centred Dynamics in Aggressive B-Cell Lymphomas

**DOI:** 10.1155/2012/138079

**Published:** 2012-02-19

**Authors:** Matilde Cacciatore, Carla Guarnotta, Marco Calvaruso, Sabina Sangaletti, Ada Maria Florena, Vito Franco, Mario Paolo Colombo, Claudio Tripodo

**Affiliations:** ^1^Dipartimento di Scienze per la Promozione della Salute, Sezione di Anatomia Patologica, Università degli Studi di Palermo, 90127 Palermo, Italy; ^2^Dipartimento di Oncologia Sperimentale, Unità di Immunologia Molecolare, IRCCS Fondazione Istituto Nazionale Tumori, 20133 Milano, Italy

## Abstract

Aggressive B-cell lymphomas share high proliferative and invasive attitudes and dismal prognosis despite heterogeneous biological features. In the interchained sequence of events leading to cancer progression, neoplastic clone-intrinsic molecular events play a major role. Nevertheless, microenvironment-related cues have progressively come into focus as true determinants for this process. The cancer-associated microenvironment is a complex network of nonneoplastic immune and stromal cells embedded in extracellular components, giving rise to a multifarious crosstalk with neoplastic cells towards the induction of a supportive milieu. The immunological and stromal microenvironments have been classically regarded as essential partners of indolent lymphomas, while considered mainly negligible in the setting of aggressive B-cell lymphomas that, by their nature, are less reliant on external stimuli. By this paper we try to delineate the cardinal microenvironment-centred dynamics exerting an influence over lymphoid clone progression in aggressive B-cell lymphomas.

## 1. Introduction

B-cell malignancies represent a heterogeneous group of diseases characterized by different biological features and clinical behaviour, the latter ranging from indolent to highly aggressive. As for most neoplasms, the natural course of B-cell malignancies is characterized by tumour progression, featured by a flow of events leading to the enhancement of proliferative and invasive capabilities, towards the establishment of a more aggressive phenotype. Even if most of the processes involved in cancer progression are inherent to the neoplastic clone, this event is, actually, the result of an articulated mechanism, which seems to require the constant crosstalk between neoplastic cells and the faulty surrounding microenvironment. An ever-increasing amount of evidences suggest that this bijective relationship is a prime determinant of cancer natural history and evolution. Much has been so far discovered about the role of tumour intrinsic mechanisms of neoplastic progression, and the focus of research has been progressively shifting toward the study of microenvironment-centred dynamics. Cancer-associated microenvironment represents a multifaceted entity, which not only provides structural support to neoplastic cells (proper stroma) but also acts as a “fertile soil” that, through humoral factors (bioactive molecules such as cytokines, chemokines, and adhesion molecules), nonmalignant cellular elements of the stroma (fibroblasts and endothelial cells) and the immune system (macrophages, mast cells, B and T lymphocytes) fosters tumour clone survival and expansion, local invasion/spreading, and escape from the immunological response.

The relative contribution of these branches of the tumour microenvironment may vary in the diverse tissues and organs in which lymphomas arise as well as in different lymphoma histotypes, yet, their relevance is proved by their influence over the disease clinical outcome.

The contribution of microenvironment to lymphoma progression has been deeply investigated in indolent lymphomas (ILs), comprising chronic lymphocytic leukaemia/small lymphocytic lymphoma (CLL/SLL), lymphoplasmacytic lymphoma (LPL), Marginal zone lymphoma (MZL), and follicular lymphoma (FL), all sharing common features such as low proliferative rate of neoplastic cells and long time to disease progression and/or treatment.

ILs are indeed characterized by a constant crosstalk with the surrounding microenvironment, which plays a role in their pathogenesis and that eventually affects several aspects of their natural history.

A prototypical example of the influence of tumour microenvironment over lymphoma progression is provided by CLL. It has been shown that CLL clones characterized by CD38 and CD49d expression, harbouring an unfavourable prognosis, are able to attract CD68+ monocytes (macrophages) at site of infiltration, by CCL3 and CCL4 chemokines synthesis.

Macrophages (Ms), recruited by the neoplastic cells, in turn, release proinflammatory mediators such as TNF, inducing upregulation of the vascular cell adhesion molecule VCAM-1 on the surrounding stroma; the following VCAM-1/CD49d binding significantly increases neoplastic cell proliferation and survival [1]. Such an interchained sequence of events involving the CLL stroma thus represents a direct link with the acquisition of a clinically appreciable aggressive pace of the disease, providing a precious insight into the potential influence of microenvironment-centred dynamics over disease course.

The pressure of immune-cell-engendered stromal changes over lymphoid clone progression can be identified in ILs other than CLL and also involving, besides Ms, other cells of the innate and adaptive immune system. Indeed, bone marrow (BM) mast cells (MCs) are commonly found in association with neoplastic BM infiltrates in patients with LPL, supporting tumour expansion through vigorous CD154-CD40 stimulation [2].

In FL, neoplastic cells benefit of the association with follicular helper T cells (Th), follicular dendritic cells (FDCs), Ms and FOXP3-expressing T regulatory cells (Tregs), for the shaping of an aberrant stromal microenvironment permissive for germinal centre (GC), neoplastic B cells [3, 4], also T helper 17 (Th17) and other IL-17-producing cells are likely to play a role in this setting.

In line with their strong reliance from lymphomatous/leukemic microenvironment, ILs show a diversified degree of tropism for stromal niches, which they colonize and subdue. Relevant examples are provided by FL cells tropism for osteoblastic/paratrabecular BM niches, rich in cellular and extracellular components (Jagged-1 Notch ligand, *β*-1integrins, type I collagen, osteopontin, and SPARC matricellular proteins) shared by the GC environment itself [5], and by splenic MZL cell homing to sinusoidal vascular niches of the BM and spleen sharing chemoattractive and adhesive signals (CXCL-12, hyaluronan, and ICAM-1) [6, 7].

The tight relation and mutual influence between neoplastic cells and their stromal microenvironment, which we have outlined for ILs, may appear less germane to aggressive lymphomas that, by their nature, show a stronger proliferative attitude and a high invasive behaviour. In these malignancies, the microenvironment role has been marginally considered in tumour progression and, therefore, poorly studied.

 The aim of this paper is to highlight the actual relevance of the stromal microenvironment in the natural history of B-cell aggressive lymphomas, trying to provide a detailed perspective of the relevant interactions involving bystander immune and mesenchymal cells and extracellular components of the stroma.

## 2. Bystander Immune Cells

**Figure 1:**
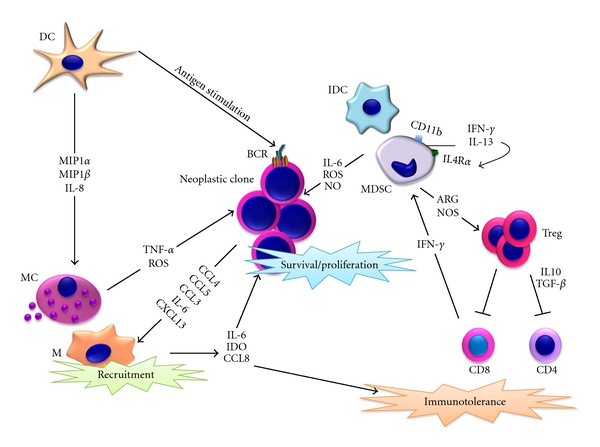
Immune system cells, both adaptive and innate, engender a fertile microenvironment through direct interaction and release of factors in the milieu. (M: monocyte/macrophage; MC: mast cell; DC: dendritic cell; IDC: immature dentritic cell; MDSC: myeloid derived suppressor cell; T reg: T regulatory lymphocyte. Black arrows indicate activation pathways. Red lines indicate inhibitory pathways).

The tumour microenvironment is populated by cells of the adaptive and innate immune system that, interacting with cancer cells, may contravene to their primary “guardian” function by actively contributing to tumour onset and progression (Figure 1).

The amount, composition, and location of both adaptive and innate immune system components vary greatly between the different types of malignant lymphoma and exert a diverse influence on the prognosis [8].

T-cell-/histiocyte-rich large B-cell lymphoma (THRLBCL) represents a paradigmatic variant in which the neoplastic large B cells constitute a minority of the tumour burden in the context of a dense microenvironment rich in T cells, with or without histiocytes [9]. Such a picture displays a high degree of homology with that of classical Hodgkin's lymphoma, in which neoplastic cells induce the recruitment of a constellation of immune cells (lymphocytes, granulocytes, Ms, DCs) from the peripheral circulation that, in turn, induce a favourable environment to the neoplasm maintenance and progression [10–12].

Tumour associated macrophages (TAMs) of THRLBCL are recruited within neoplastic infiltrates mainly by clone-derived macrophage chemotactic proteins (MCPs) and represent a major component of the infiltrate. Within the cancer-associated microenvironment, TAMs display a peculiar dual-faceted attitude; they can kill tumour cells but at the same time favour their growth by inducing immunosuppression and producing angiogenic factors and metalloproteases [13]. In most aggressive B-cell lymphomas, TAM protumoral function neatly prevails over their participation to antitumour immunity. Specifically, in THRLBCL IFN-*γ*-induced TAM activation determines the synthesis of the chemoattractant protein CCL-8 (MCP-2) and of the immunomodulatory molecule indoleamine 2,3-dioxygenase (IDO), that give rise to a self-feeding immunosuppressive loop [14].

**Figure 2:**
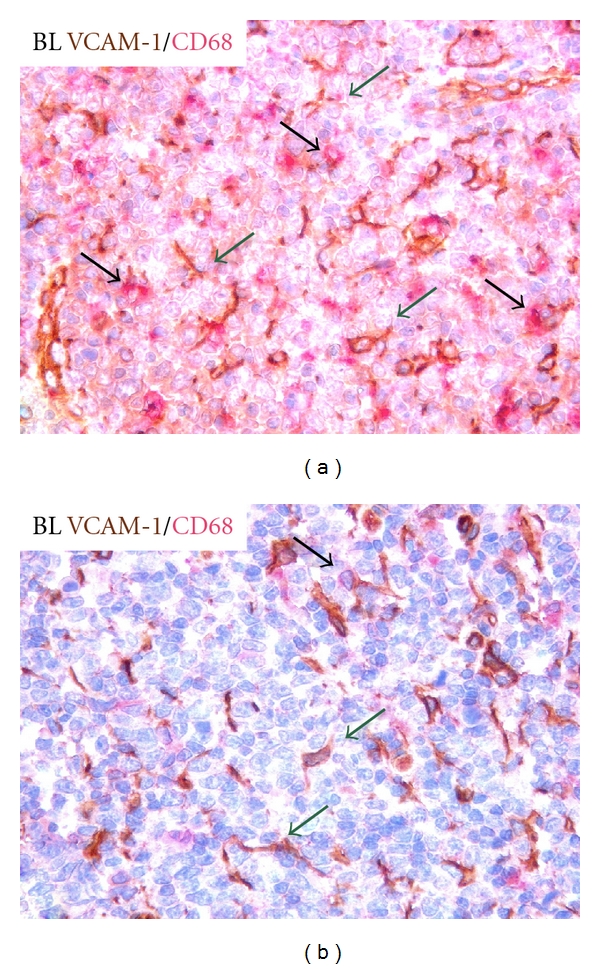
Adhesion molecule VCAM1-expressing mesenchymal cells (green arrows) form a denser meshwork in BL cases rich in CD68-expressing macrophages (black arrows) as compared with cases showing scattered CD68-expressing macrophages. (VCAM and CD68 immunohistochemical stain performed with streptavidin-biotin peroxidase complex system, original magnification 400x).

A significant M infiltration is observed in other aggressive B-cell lymphomas such as in Burkitt's lymphoma (BL), in which Ms are functionally involved in neoplastic apoptotic cell engulfment and are stimulated by IL-10 to the synthesis and release of B-cell trophic factors such as BAFF/BLyS [15]. In addition to directly stimulate neoplastic B cells, CD68-expressing Ms induce activation of the neighbouring mesenchymal cells as demonstrated by VCAM-1 upmodulation in areas of prominent M infiltration (Figures 2(a) and 2(b)).

Similarly, in diffuse large B-cell lymphoma (DLBCL), neoplastic cells recruit T cells and CD14+ monocytes by CCL-5 release, also engendering a histiocyte-enriched microenvironment [16]. Besides local synthesis of soluble mediators active on neoplastic B cells, infiltrating Ms and other professional antigen-presenting cells (APCs) can support neoplastic B-cell proliferation and rescue from apoptosis by sustained B-cell-receptor (BCR) stimulation [17].

BCR signalling pathway can be triggered in neoplastic cells by canonical antigen ligation or by antigen-independent adhesive signals modifying the actin cytoskeleton and in both cases involves the activation of the Syk kinase [18]. BCR stimulation by environment-generated signals can be relevant for the fitness of neoplastic B cells, yet, in several aggressive B-cell lymphomas, as in DLBCL, constitutive activation of the BCR pathway (i.e., tonic signalling) can be observed, which is not dependent on external stimuli [19, 20]. On these bases, inhibition by Syk targeting, irrespectively of the neoplastic cell-intrinsic or cell-extrinsic source of BCR stimulation, could be envisaged as an appealing therapeutic prospect [21].

Among monocytic/macrophagic CD14-expressing cells recruited by DLBCL clones, intratumoral precursors of dendritic cells (DCs) have been identified basing on their expression of the DC marker DC-SIGN and on the acquisition of DC morphology. DCs found within tumour infiltrates are commonly “frozen” in an immature status (iDCs) by soluble factors of the tumour milieu such as IL-4, IL-6, GM-, and M-CSF. iDCs are also recruited from the BM myeloid cell reservoir through CCL-3 and CCL-4 chemokine interaction with CCR-1/-2/-5 receptors and on their turn participate to the recruitment of other myeloid cells at sites of infiltration (e.g., by IL-8, RANTES, TARC, and MDC) [22, 23]. Among BM-derived myeloid cells that might be coopted by neoplastic B cells or bystander cells in the lymphoma-associated microenvironment, a relevant population is represented by myeloid-derived suppressor cells (MDSCs). With iDCs, MDSCs share an immature myeloid phenotype and are characterized by the expression of the CD11b, CD33, and IL4r. Both iDCs and MDSCs empower the regulatory milieu associated with the expanding clone by the inhibition of T-cell responses through nitric oxide (NO) and reactive oxigen species (ROS) release and induction of Treg skewing. It is conceivable that the restoration of the physiologic crosstalk between DCs (or other APCs) and T cells, or the functional inactivation of such myeloid regulatory cells [24, 25], might induce effective Th-1-oriented cytotoxic responses against the B-lymphoid clone. Indeed, a dense cytotoxic T-cell infiltrate spatially associated with CD21-expressing FDC meshwork has been correlated with better survival and a higher complete remission rate in high-risk DLBCL [26], thus suggesting a favourable influence of effector T cells populating the lymphoma environment. However, other studies have shown that dense infiltrates of activated cytotoxic T cells in nodal DLBCL correlate with poor survival [27] indicating that the actual outcome of T-cell infiltration is indeed puzzling and variable.

If the interpretation of the contribution of infiltrating T cells to the composition of the lymphoma microenvironment appears rather problematic, this is particularly true for Tregs [4, 28, 29]. In DLBCL, the prognostic significance of infiltrating Tregs has proved quite controversial, since the amount of Tregs associated with lymphoma infiltrates has been found to independently correlate with a good [30, 31] or dismal prognosis (or found unrelated with prognosis) [28, 31] by different Authors. The controversial results of Treg prognostic influence in DLBCL has been interpreted in light of the heterogeneity of settings and methods adopted for Treg assessment [28], yet they probably have a true biological explanation.

Tregs modulate the activity of both CD4+ and CD8+ effector populations through the release of IL-10 and TGF-*β*1. Therefore, they can contribute to the immune escape of the neoplastic clone producing a detrimental influence on outcome while, at the same time, depriving neoplastic cells from beneficial proinflammatory stimuli induced by other lymphoma-infiltrating cells. Moreover, the above-mentioned analyses of Treg influence in the lymphoma-associated environment are intrinsically flawed by the assumption that Tregs are “functionally stable” which means not considering their plasticity. In their activity of quenching immune responses by interfering with the activation status of innate (e.g., MCs) and adaptive (e.g., T cells) immune effectors, Tregs are exposed to the pressure of the inflammatory milieu, by which they can be skewed towards other functional fates. We have recently demonstrated that activated MCs can induce contrasuppression of Tregs through the OX40/OX40L axis and IL-6 release in a TGF-*β*1*-*rich environment towards the generation of proinflammatory Th17 cells [32, 33]. In this light, the different prognostic value of FOXP3+ Tregs in GC-type DLBCL where their presence is related with a positive prognostic influence and non-GC DLBCL where, by contrast, an increase of FOXP3+ Tregs directly correlates with an adverse outcome, could be at least partially explained by the marked inflammatory environment engendered in the latter by the abundancy of IL-6- and TNF-producing Ms and MCs [34–36]; this could inhibit the function of Tregs which, in turn, could even boost inflammation favouring Th17 generation [28].

Overall, it is hard to identify a *leitmotiv* in the functional interactions between neoplastic cells and immune bystander cells of aggressive B-cell lymphomas as the final outcome of such interplay can profoundly vary at discrete stages of the disease course and can be significantly affected by therapy. Nonetheless, aggressive B-cell lymphoid clones, despite their striking proliferative and invasive capabilities, are not disengaged by the influence of the immune microenvironment, which actually represents a reasonable focus for chasing the improvement of treatment efficacy.

## 3. The Matter of Vasculogenesis

Among the different aspects functional to tumour progression characterizing the cross talk between neoplasms and stroma, a remarkable role is played by vasculogenesis. Neo-angiogenesis has been the focus of extensive scientific investigation in the field of cancer research. The generation of new blood vessels not only provides a dedicated blood supply to the tumour, but also represents the hinge of its dissemination, being the most direct route for the colonization of secondary organs.

**Figure 3:**
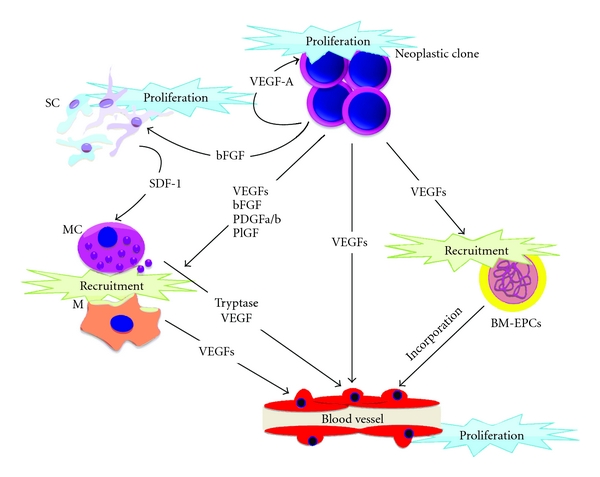
Vasculogenesis plays a key role in clone dissemination and represent a the route for organ colonization. (M: monocyte/macrophage; MC: mast cell; SC: stromal cell; BM-EPC: bone marrow derived endothelial progenitor cell. Black arrows indicate activation pathways).

In aggressive B-cell lymphomas, as in several other cancers, Neo-angiogenesis is the result of a play of forces between neoplastic and stromal elements involving the axis of vascular endothelial growth factors (VEGFs) and their receptors, known to play a central role in this process (Figure 3). The synthesis of VEGFs along with the expression of their receptors has been extensively described in DLBCL [37], Mantle cell lymphoma (MCL) [38], BL [39], and grade-3 FL [40], being reported as variably associated with the disease aggressiveness.

In such B-cell lymphomas, neoangiogenesis is regulated by an intricated and redundant network of interactions based on the production of growth factors deriving from malignant cells and from accessory cells of the microenvironment. Production of VEGFs (VEGF-A, VEGF-C, VEGF-D) and other angiogenic factors such as the basic fibroblasts growth factor (bFGF), placental growth factor (PlGF), platelet-derived growth factors (PDGF-a and PDGF-b) and stromal-derived factor-1 (SDF-1) by cancer and stromal cells initiates neoangiogenesis through the contemporary involvement and recruitment of different cytotypes [41]: mature resident endothelial cells receiving direct mitogenic signals stemming from Raf-1-MEK-MAP kinase cascade after interaction with VEGFs [42]; CD68+ monocyte-macrophages and other accessory cells such MCs [43, 44], further enriching the microenvironment by the release of bFGF, PDGF, members of the VEGF family, proteases (e.g., MC-tryptase, matrix metalloproteases), and proinflammatory mediators (IL-1, IL-6, IL-8, and TNF) [45–47]; BM-derived endothelial progenitor cells and BM mesenchymal stem cells recruited by VEGF and SDF-1 gradients, amplifying the synthesis of proangiogenic mediators and eventually incorporating into the growing tumour neovasculature [5, 48–50]. Furthermore, neoplastic B lymphocytes directly perceive pro-angiogenic stimuli through the expression of VEGF receptors (VEGFR-1 and VEGFR-2), which enables them to receive, in an autocrine fashion, proliferation and/or survival signals [50]. A high expression of VEGF therefore directly links the remodelling of the stromal vascular microenvironment to clone-intrinsic B-cell lymphoma progression. Several studies have been so far focused on the relationship between microvascular density (MVD) of lymphomatous infiltrates, prognosis, and clinical outcome in B-cell lymphomas; in general, MVD scores trend higher in aggressive histotypes including BL and DLBCL, compared with indolent ones [51].

The correlation between MVD and the clinical course of different lymphoma subtypes, however, is not straightforward. In DLBCL, for instance, multiple studies have demonstrated that there are no significant differences in the MVD counts between the long- and short-surviving patients and that MVD score does not correlate with overall survival [52, 53]. Conversely, other experimental evidences have suggested a negative impact of vasculogenesis on DLBCL clinical outcome by demonstrating that MVD increases paralleling tumour progression [46, 54].

Differently from DLBCL, in BL the degree of neoangiogenesis is more homogeneously related to the disease progression. BL aggressive behaviour depends on the overexpression of the oncogene *MYC*, which also acts as an essential promoter of early and persistent growth of blood and lymphatic vessels during tumour progression [55]. Moreover, as demonstrated in a murine model of BL, c-MYC-expressing B cells are the major source of the vascular endothelial growth factor [56].

The mechanism through which c-MYC regulates the VEGF axis has not yet been clearly elucidated. c-MYC may conceivably act as a VEGF transcriptional factor [57], and recent data suggest that it could control the expression of several mRNAs (such as the one encoding for VEGF) by regulating a broad range of microRNAs [58].

Along with BL, also MCL is characterized by a strong and active neoangiogenetic process. In MCL, neoplastic B cells give rise to an autocrine positive feedback mediated by the VEGF-A/VEGFR-1 axis, which is also sensed by stromal cells of blood and lymphatic vessels expressing VEGFR-2 and VEGFR-3 [59].

The established leading role played by neoangiogenesis in aggressive B-cell lymphomas paved the way to several clinical trials targeting the formation of new blood and lymphatic vessels. Both consolidated and ongoing approaches are based on the administration of different combinations of drugs capable to interfere with the VEGF axis either in a direct or an indirect manner. Examples of these strategies are the use of anti-VEGF-A antibodies (e.g., bevacizumab) in association with chemoimmunotherapy, and the administration of endostatin and anti-CXCR-4 monoclonal antibodies in the prospect of blocking circulating endothelial cell progenitors [60, 61].

Neoangiogenesis is intimately involved in the arousal and progression of B-cell neoplastic clones but dissecting its relative contribution to these processes is problematic owing to the strong correlations it displays with almost every other microenvironment-centred process. This apparent limit to the understanding of the true influence of angiogenesis in the setting of aggressive B-cell lymphomas actually represents a precious advantage for antiangiogenic treatments, which are able to interfere with multiple vicious dynamics of the lymphoma-associated microenvironment, including recruitment of accessory cells, recruitment, and integration of mesenchymal and endothelial progenitors as well as with neoplastic B-cell dissemination.

## 4. The Extracellular Matrix: More Than Scaffolding

Extracellular matrix (ECM) has been considered, for many years, an inert scaffold composed by a complex mixture of proteins, proteoglycans, and in some cases of bone mineral deposits, aimed at providing support and anchorage to cells and regulating intercellular communication.

Synthesized by stromal cells, the ECM represents a reservoir for many growth factors and can be digested by enzymes like serine and threonine proteases and matrix metalloproteases to favour homeostatic processes like tissue remodelling and repair [62].

Similarly, the tumour-associated microenvironment undergoes continuous remodelling, and the ECM components, produced and released by tumour and nonneoplastic stromal cells, represent a major vehicle for the tumour-stroma crosstalk. Accordingly, ECM components have been implicated in tumor growth, progression, and metastasis both in solid and lymphoid malignancies [63–66].

One notable attempt to investigate the influence of the ECM on aggressive lymphoma behaviour has been performed in the DLBCL setting [65] following the identification of different DLBCL prognostic categories based on the expression of tumour-related genes [67]. The study performed by Lenz et al. [65] explored DLBCL from a stromal perspective and highlighted a group of cases showing a “stromal signature” enriched in ECM genes coding for collagens, laminin, metalloproteases, and matricellular proteins. This signature was related with a more favourable prognosis as compared with that of another group of DLBCL cases that was enriched in genes promoting the “angiogenic switch.”

Matricellular proteins are a class of nonstructural ECM proteins endowed of regulatory function during tissue remodelling [68] and cancerogenesis [69]. Among them, SPARC (secreted protein acidic rich in cysteine), thrombospondins, and osteopontin (OPN) have been reported to play a pivotal role in providing proliferative and antiapoptotic signals to cancer cells, influencing their binding to structural matrix components or directly triggering tumour cell surface receptors [70]. Through the engagement of specific integrin receptors or CD44, OPN exerts its pleiotropic function in cancer cell survival, ECM remodelling, cell migration, and metastasis in solid cancers as well as in aggressive B-cell lymphomas [71–75]. One of the mechanisms hypothesized as responsible for the promotion of neoplastic cell survival by OPN relies on its binding to CD44. OPN-mediated CD44 engagement can prevent cell death by activation of the phosphatidylinositol 3-kinase/Akt signaling axis and by inhibition of Fas-induced signals [76, 77]. In breast cancer, OPN is also able to modulate the expression of specific CD44 isoforms [78] such as the CD44v6 and v9 variants, which endorse a negative prognostic significance. Tissue microarray analyses performed in DLBCL cases, demonstrated that the expression of CD44v6 variant was predominant in the activated type of DLBCL and, in CD44 negative cases, correlated with a worse prognosis [79].

Besides interacting with CD44 and integrins expressed on neoplastic cell surface, OPN contributes to moulding the cancer-associated immunological microenvironment by directly inducing Ms recruitment and activation towards amplification of the inflammatory milieu rich in TNF, IL-1b, and IL-6 [80, 81]. These dynamics, which have been extensively investigated in many solid tumours like soft tissue sarcomas [82] and breast cancer [83], could also take place in haematological malignancies owing to the critical role played by OPN in regulating normal and aberrant hematopoiesis [84].

**Figure 4:**
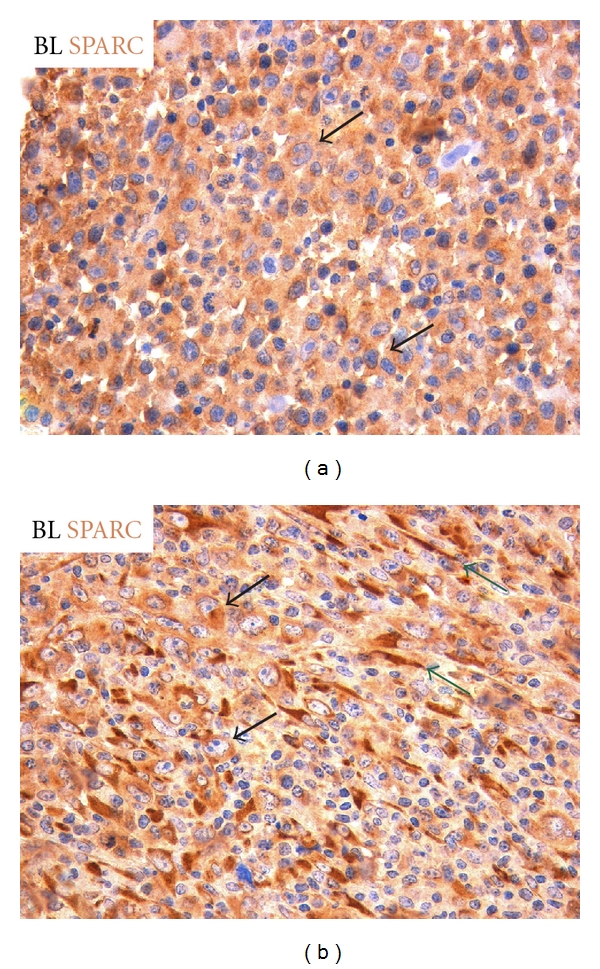
SPARC immunohistochemical analysis of BL cases reveals rather homogeneous expression in neoplastic cells (black arrows) in spite of a variable stroma SPARC reactivity (green arrows). (SPARC immunohistochemical stain performed with streptavidin-biotin peroxidase complex system, original magnification 400x).

Another matricellular protein, whose multifaceted influence in cancer microenvironment has been progressively delineating, is SPARC. SPARC, also known as osteonectin or BM-40, is a secreted, matricellular glycoprotein exerting an homeostatic function in tissue remodelling, being capable of regulating biological processes like angiogenesis, cell proliferation, collagen deposition, and inflammation [85]. The tissue-normalizing function of SPARC could be extended to cancer with implications for tumour growth, invasion, and metastasis. However, SPARC expression and functions are greatly tissue and context dependent, and their investigation often ingenerates ambiguous results. For example, in some solid cancers, conspicuous SPARC expression by neoplastic and stromal cells can either promote epithelial-to-mesenchymal transition (EMT), and favour tissue invasion and metastasis, or exercise an antiproliferative effect on neoplastic cells, thus limiting cancer progression [86, 87]. Actually, SPARC function in the tumour microenvironment is deeply influenced by its cellular source. SPARC produced by stromal and immune cells may exert diversified influences over the neoplastic clone, the former contributing to cancer stromatogenesis and stromal remodelling while the latter normalizing the inflammatory milieu by negatively regulating immune cell infiltration and activation (e.g., through suppression of the NF-kB pathway) [75, 88]. SPARC has a paramount importance in the regulation of structural ECM composition [85], in which it participates as a collagen chaperon. The valency of SPARC in regulating the stromatogenesis triggered by neoplastic clones has also a considerable degree of ambiguity. Indeed, SPARC is required for the correct assembly of the collagen meshwork that provides adhesive substrate to cancer cells, yet, it might also inhibit integrin-mediated adhesion and the generation of signals stemming from the integrin-linked kinase activation [89]. Recently, some of us have reported that *SPARC* gene is highly expressed as part of the GC-related signature of BLs, where it specifically characterizes the endemic BL (eBL) subgroup [90]. Notably, SPARC-, TGF-*β*-, and other EMT-inducer-derived signals, including those stemming from Notch receptors, converge at the Ras-MAPK pathway, which was found upregulated in BLs in spite of a biased BCR signal initiation [90]. This picture is in line with the cellular program of BL oriented towards proliferation, migration, and ECM invasion, and poorly reliant on extracellular signals. SPARC protein expression in BL samples consistently marked neoplastic cells but also variably characterized stromal cells of the microenvironment (Figure 4) suggesting a potential involvement of this molecule in stroma-centred dynamics of BL and other GC-associated neoplasms, which haven't been so far explored [90]. In this regard, a role for SPARC produced by FDCs in orchestrating GC T-cell trafficking towards the establishment of Th-17-mediated responses has been recently demonstrated [91].

Besides Osteonectin and SPARC, many other molecules take part to the complex network created by neoplastic cells and ECM components. In BL cell lines, it has been demonstrated that the ECM protein fibronectin, following binding of alphavbeta3-integrin expressed on neoplastic cell surface, activates signal transduction pathways leading to BL cell proliferation by phosphorylation of the MAP kinase ERK-2 [92]. Similar interactions between integrins and multiple ECM binding partners, namely, vitronectin, laminin, type I and type IV collagen, have been reported to occur in different solid cancer settings and can be also predicted in aggressive lymphomas [93–96].

Matrix metalloproteases (MMPs) were at first identified as mere ECM-regulating components but their involvement in the interplay with factors other than ECM derived, such as growth factors and their receptors, cytokines and chemokines, adhesion receptors, cell surface proteoglycans, and a variety of enzymes has progressively come into evidence [97, 98]. The expression and production of different MMP subtypes in aggressive B-cell lymphomas may depend not only on the different biology of the neoplastic clone, but could be also determined by the surrounding environment [99]. IL-6 produced by reactive lymphocytes, Ms, endothelial cells, and fibroblasts induces MMP-9 and MMP-2 production that, in aggressive B-cell lymphomas, may lead to a more aggressive clinical behaviour [100].

IL-6 produced in the lymphoma microenvironment also acts as a positive regulator of tissue inhibitor of metalloproteinase (TIMP) expression by neoplastic and stromal cells. TIMPs are capable of inhibiting the activity of MMPs thus keeping the balance between ECM deposition and degradation processes; however, multifaceted and apparently paradoxical actions of TIMPs (i.e., TIMP-1 and TIPM-2) have been recently reported, suggesting their direct contribution to lymphoma progression [101].

In fact, TIMP-1 produced by neoplastic B lymphocytes, fibroblasts, and endothelial cells [102] has been shown to inhibit germinal centre B-cell apoptosis and promote cancer cell survival in aggressive B-cell lymphomas. The antiapoptotic effect of TIMP-1, which could be considered one of the causes contributing to the poor survival evidenced in some of these cases, is not to ascribe merely to MMP inhibition and/or cell-matrix interactions, but also to the binding of TIMP-1 to other cell-surface receptors, independent of MMP inhibitory function [103].

In BL cells in which TIMP-1 promotes postgerminal centre B-cell differentiation by upregulating MUM-1 and CD138 and downregulating BCL6, its overexpression leads to the activation and expression of STAT3, and to the upregulated expression of cyclin D2, CD44 and BCL-XL, the latter being a target protein of STAT3 with prominent anti-apoptotic function [104].

**Figure 5:**
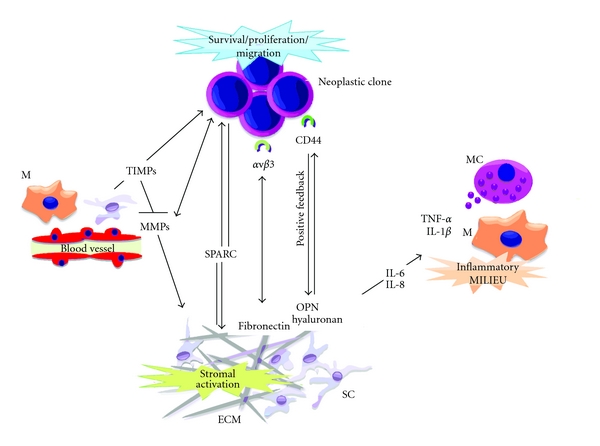
Extracellular matrix orchestrates a functional network through its structural and secreted elements. (M: monocyte/macrophage; MC: mast cell; SC: stromal cell; ECM: extracellular matrix. Black arrows indicate activation pathways. Red lines indicate inhibitory pathways).

ECM thus emerges as much more than inert scaffolding for lymphomatous cells, representing a major source of direct “rescue” signals and also critically influencing several aspects of the lymphoma-associated environment, such as the trafficking and activation of immune cells (Figure 5). Although strategies aiming at inducing modifications in the ECM components are hardly plausible, owing to the elevated redundancy of the cellular dynamics leading to ECM regulation, ECM should be considered as a precious source of information regarding the biology of the underlying neoplasm, and ECM-related cues (such as miRNAs) should be taken into account as potential cancer-related markers for risk stratification and prognostication [105].

## 5. Conclusions

By delineating the main microenvironmental dynamics that take place in aggressive B-cell lymphomas, we aimed to convey the message of a leading role played by the nonneoplastic lymphoma-associated immunological and stromal elements in influencing the natural history of these highly malignant neoplasms, so far classically considered poorly reliant on the environment. The mutual influence between neoplastic B lymphocytes and their microenvironment results in the enhancement of the proliferative and invasive capabilities of the neoplastic clone and in the concurrent reshaping of the infiltrated tissues.

A deeper understanding of such relationship, through the dissection of its complex dynamics, could prove a successful enterprise for the establishment of multitargeted therapeutic approaches and for the identification of new prognostic factors reflective of the clone-extrinsic biology of these B-cell lymphomas.
